# Mo@ZIF-8 nanozyme preparation and its antibacterial property evaluation

**DOI:** 10.3389/fchem.2022.1093073

**Published:** 2022-11-24

**Authors:** Zheng Lian, Chunqing Lu, Jiangqi Zhu, Xining Zhang, Ting Wu, Youlin Xiong, Zhiyi Sun, Rong Yang

**Affiliations:** ^1^ School of Criminal Investigation, People’s Public Security University of China, Beijing, China; ^2^ CAS Key Laboratory for Biomedical Effects of Nanomaterials and Nanosafety, Center of Materials Science and Optoelectronics Engineering, CAS Center for Excellence in Nanoscience, National Center for Nanoscience and Technology, University of Chinese Academy of Sciences, Beijing, China; ^3^ Institute of Evidence Law and Forensic Science, China University of Politic Science and Law, Beijing, China

**Keywords:** peroxidase-like nanozyme, metal organic framework, ZIF-8, molybdenum, antibacterial therapy

## Abstract

Types of nanozymes can produce free radicals and/or reactive oxygen species (ROS) to serve as broad spectrum antibacterial materials. Developing nanozyme-based antibacterial materials with good biocompatibility exhibits promising application prospects. In this study, we doped Mo to ZIF-8 (both components have good biocompatibility) to prepare a new nanozyme, Mo@ZIF-8, which can produce hydroxyl radicals (•OH) triggered by a low dosage of hydrogen peroxide (H_2_O_2_), exhibiting effective antibacterial capability against both Gram-negative bacteria (*Escherichia coli*) and Gram-positive bacteria (*Staphylococcus aureus*). This work provides a reference for the design of antibacterial nanozymes with good biocompatibility.

## 1 Introduction

Bacterial-related diseases has been one of public safety issues that greatly threaten the health of humankind. (Reverter et al., 2020; Yu et al., 2022; Ding et al., 2022). Antibiotics have been the most widely accepted treatment for bacterial infection diseases. (Stracy et al., 2022). However, the abuse of antibiotics attribute to the emergence of drug-resistant bacteria, which may threaten the global health and environment. (Lakemeyer et al., 2018; Serwecińska, 2020). Therefore, effective and broad-spectrum antibacterial agents are urgently needed.

Currently, types of materials, such as metals (Gu et al., 2020; Tian et al., 2021), metal oxides (Li et al., 2018; Dong et al., 2022), carbon materials (Bi et al., 2022), single-atom materials (Cai et al., 2022) and MXenes (Yu et al., 2022b), have been verified to have peroxidase (POD)-like activity when they are fabricated to nanoscale. These nanozymes can convert H_2_O_2_ into OH radicals to effectively kill bacteria.

Metal organic frameworks (MOFs) have large surface area and pore structures, which provides scaffold for the enzymatic (catalytic) performance. (Ma et al., 2020). Here, we developed a new type of antibacterial nanozyme (Mo@ZIF-8) by doping Mo to ZIF-8. Briefly, the prepared hexahedron-shaped ZIF-8 (150 nm) was refluxed with Na_2_MoO_4_ solution and subsequent pyrolyzed at 600°C. The prepared Mo@ZIF-8 exhibited promising capability producing •OH and killing both Gram-negative (*E. coli*) and Gram-positive (*S. aureus*) bacteria at a low dosage of H_2_O_2_ (10^−5^ M). Due to the high biocompatibility of Mo and ZIF-8, this rationally designed nanozyme has the potential to be an effective antibacterial agent.

## 2 Experimental

### 2.1 Synthesis of hollow Mo@ZIF-8 nanostructures

0.595 g Zn(NO_3_)_2_·6H_2_O was dissolved in 20 mL methanol (solution A). 0.656 g 2-methylimidazole (2-MIM) was dissolved in 20 mL methanol (solution B). Then solution B was rapidly added into solution A under vigorous for 15 min stirring at room temperature. The mixed solution was stand for 3 h at room temperature. After washing with methanol for three times, the white powder was collected by centrifugation and dried at 40°C.

To prepare Mo@ZIF-8 nanostructures, 0.075 g of as-prepared ZIF-8 was dispersed in 30 mL ethanol. Then 15 mL of Na_2_MoO_4_ solution (containing 0.0375 g Na_2_MoO_4_·2H_2_O) was mixed with ZIF-8 solution, then the mixed suspension refluxed at 82°C for 2 h. The precipitation was collected and wash by methanol for three times. The product was dried at 40°C and further annealed in Argon atmosphere at 600°C for 2 h with the rate of 5°C/min.

### 2.2 Enzyme-like activity and catalytic kinetics studies

The peroxidase-like activity of Mo@ZIF-8 nanostructures was tested by 3,3′,5,5′-tetramethylbenzidine (TMB) as substrate in the presence of H_2_O_2_. First, the Mo@ZIF-8 catalysts were dispersed in water with ultrasonication. Then, 1 mg/mL (50 µl) suspension was added to 450 µl NaAc–HAc buffer (0.1 M pH 3.0) containing 1 mM TMB and 2 mM H_2_O_2_. After incubation at room temperature for 20 min, the UV-vis absorption of the mixture was recorded. The influence of pH (2–9) and temperature (20–80°C) on the catalytic performance of Mo@ZIF-8 were also evaluated.

The steady-stated kinetic experiments were carried out in 500 µL NaAc–HAc buffer (0.1 M pH 3.0) containing 100 μg/mL Mo@ZIF-8, 1 mM TMB and H_2_O_2_ ranging from 0 to 2.0 mM, or containing 100 μg/mL Mo@ZIF-8, 2 mM H_2_O_2_ and TMB ranging from 0 to 2.0 mM. The absorbance changes at 652 nm were constantly monitored in time-scan mode. The kinetic parameters were determined by the following equation:

$$\frac{1}{v}=\left(\frac{K_{m}}{V_{\max}}\right)\left(\frac{1}{\left[S\right]}\right)+\frac{1}{V_{\max}}$$ (1)

where v was the initial velocity, V_max_ was the maximal reaction velocity, K_m_ was the Michaelis–Menten constant and [S] was the substrate (TMB or H_2_O_2_) concentration, respectively.

### 2.3 Antibacterial experiments

Gram-positive *S. aureus* and Gram-negative *E. coli* were used for the antibacterial experiments. Typically, the experiments for each bacterium were divided into four groups: 1) bacteria, 2) bacteria + H_2_O_2_, 3) bacteria + Mo@ZIF-8, 4) bacteria + Mo@ZIF-8 + H_2_O_2_. The concentration of H_2_O_2_ used in the process was 10^−5^ M and the concentration of Mo@ZIF-8 was 2, 5 and 10 μg/mL. The bacteria were incubated with above solution for 4 h, then 30 μl of the suspension (1.0 × 10^7^ CFU/mL *S. aureus* or *E. coli*) were spread on Luria-Bertani (LB) solid medium. These plates were kept at 37°C for 18 h, and bacterial colonies were counted.

Scanning electron microscopy (SEM) was used to observe the morphological change of the bacteria after antibacterial experiments. Groups: 1) control (bacteria without any treatment), 2) 10^−5^ M H_2_O_2_, 3) 5 μg/mL Mo@ZIF-8, 4) 5 μg/mL Mo@ZIF-8 + 10^−5^ M H_2_O_2_. After incubated for 4 h, the bacterial suspensions were collected and dropped onto silicon slides. Subsequently, bacterial cells were prefixed with 2.5% glutaraldehyde for 4 h, and dehydrated by a graded series of ethanol (30%, 50%, 70%, 80%, 90%, and 100%, respectively). The bacteria were finally dried for observation by scanning electron microscope.

## 3 Results and discussion

### 3.1 Structure characterizations

**FIGURE 1:**
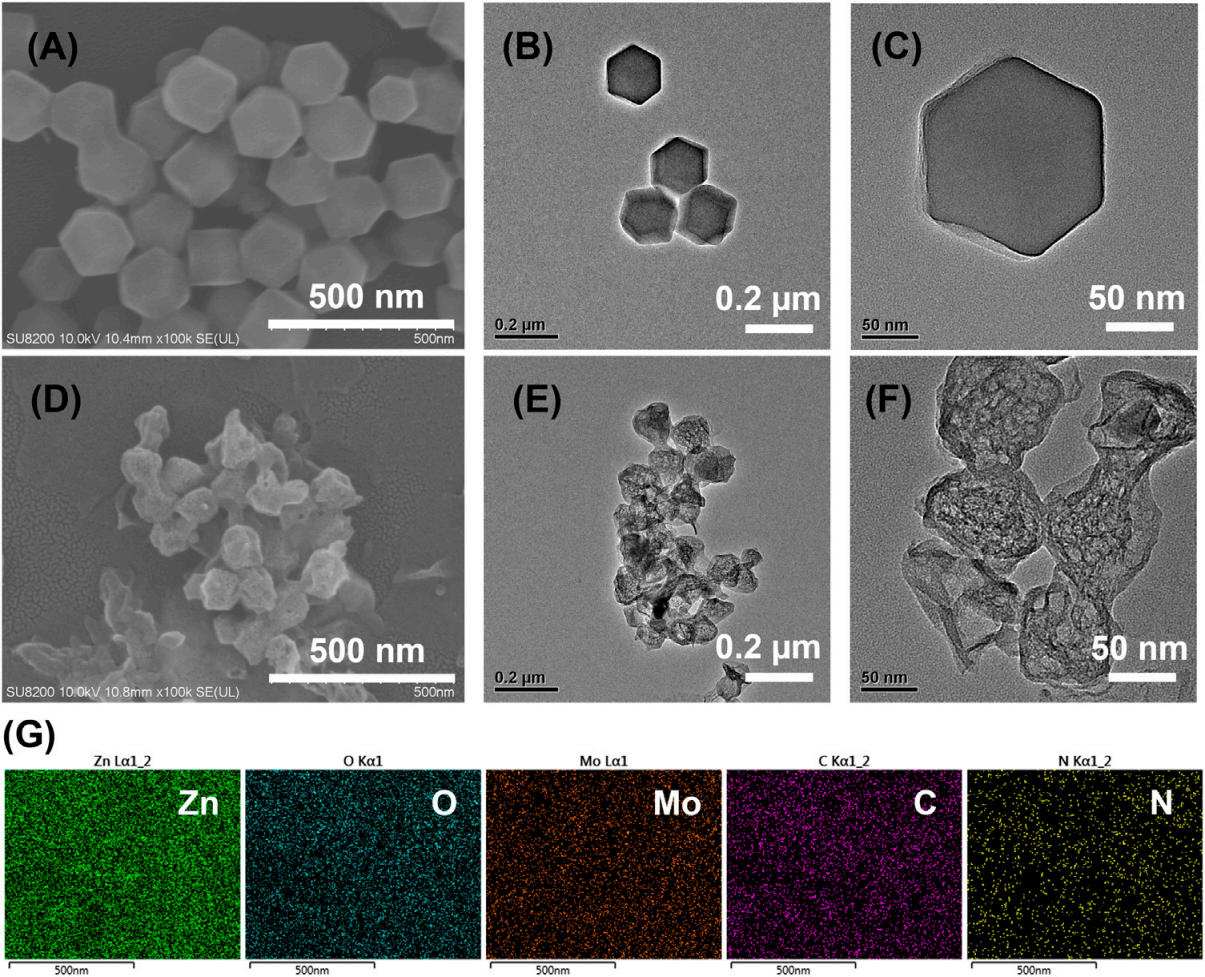
Characterizations of nanostructures from electron microscopy technique. **(A)** SEM image of ZIF-8. **(B,C)** TEM images of ZIF-8 at different magnifications. **(D)** SEM image of Mo@ZIF-8. **(E,F)** TEM images of Mo@ZIF-8 at different magnifications. **(G)** EDX analysis of Mo@ZIF-8. The images indicated that Mo had been doped to the nanostructures.

The SEM and TEM characterizations showed a hexahedron shape for ZIF-8 with the size of about 150 nm. (Figures 1A–C). Meanwhile, TEM images showed the smooth surface of the ZIF-8 crystals (Figures 1B,C). After refluxing with Na_2_MoO_4_, the product became rounded and the corner disappeared. The size of the product was smaller than ZIF-8, which was about 100 nm. (Supplementary Figure S1A,B). The product was annealed at 600°C in Argon atmosphere and Mo@ZIF-8 formed. The Mo@ZIF-8 exhibited rough surfaces and hollow structures, shown in Figures 1D–F. TEM revealed their hollow and spongy structure, and the average diameter of the Mo@ZIF-8 was about 100 nm. (Figure 1F).

The incorporation of Mo element into ZIF-8 was confirmed by Energy Dispersive X-Ray Spectroscopy (EDX). The element distribution of Mo@ZIF-8 was shown in Figure 1G, demonstrating a homogeneous distribution of Zn, O, Mo, C, N elements in the nanocomposites.

**FIGURE 2:**
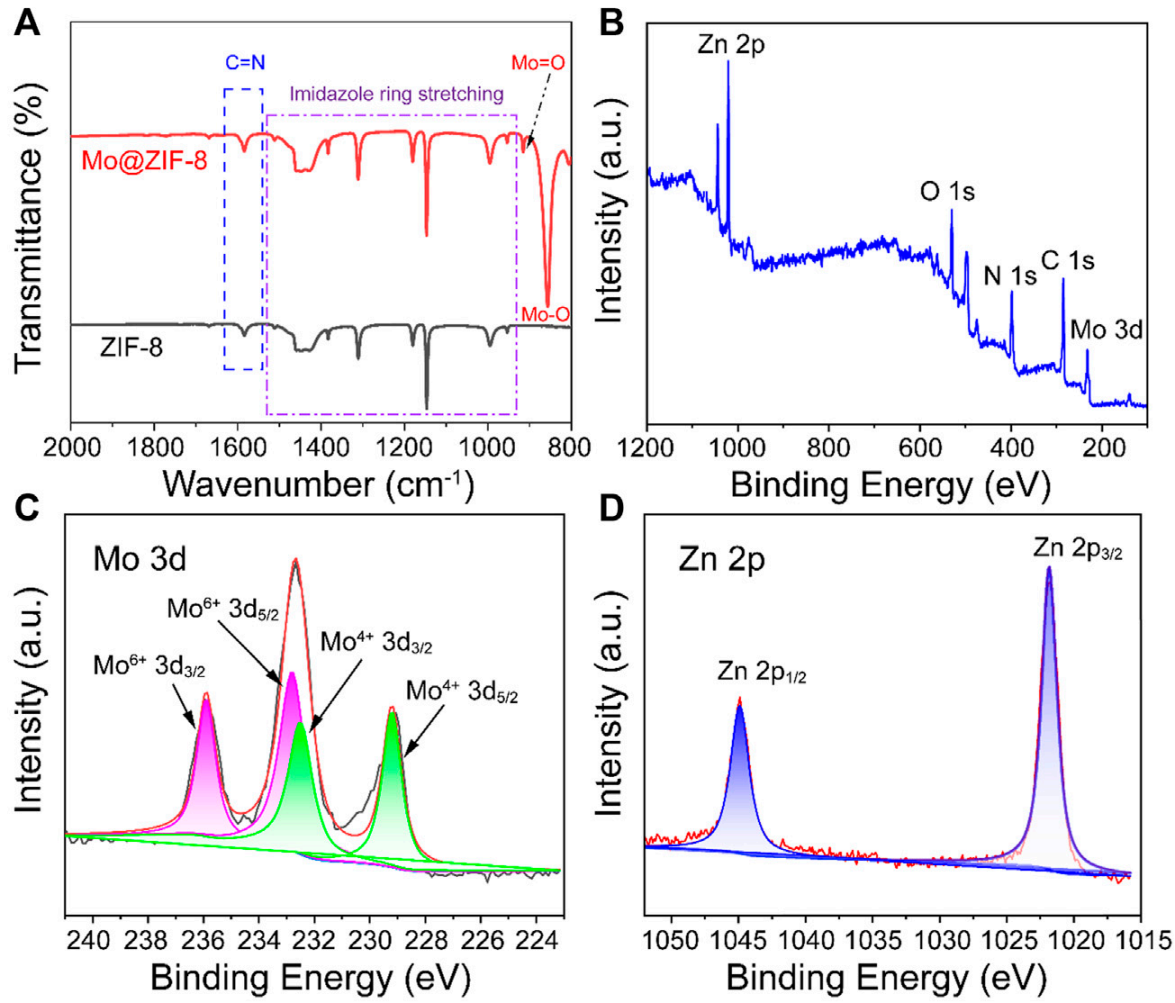
Characterizations of Mo@ZIF-8. **(A)** The FT-IR spectra of ZIF-8 and Mo@ZIF-8. The stretching peaks in the ZIF-8 and Mo@ZIF-8 were labeled. **(B–D)** XPS spectra of Mo@ZIF-8. **(B)** Survey spectrum. High-resolution XPS spectra of **(C)** Mo 3d, **(D)** Zn 2p.

FT-IR spectra of ZIF-8 and Mo@ZIF-8 were performed to demonstrate the successful incorporation of Mo, as shown in Figure 2A. The FT-IR spectra of ZIF-8 and Mo@ZIF-8 exhibited the characteristic peaks of 953–1,511 cm^−1^ and 1,582 cm^−1^, which corresponded to the signals of the imidazole ring stretching and C=N bond of ZIF-8. (Wang et al., 2016; Zhang and Park, 2019). This revealed that the introduction of Mo did not destroy the imidazole ring. For the Mo@ZIF-8, new peaks appeared at 857 cm^−1^ and 916 cm^−1^, which might be attributed to the stretching vibration of Mo-O and Mo=O bond, respectively. (Lin et al., 2020).

Furthermore, X-ray photoelectron spectroscopy (XPS) was explored the chemical composition and element valence state of Mo@ZIF-8 nanocomposites. Wide-scan XPS spectrum (Figure 2B) indicated the presence of C, O, N, Zn, and Mo in the Mo@ZIF-8 nanocomposites, confirming that Mo was successfully loaded on ZIF-8. Figure 2C was the high-resolution XPS spectrum of Mo 3d. Three bands at 235.9 , 232.6, and 229.2 eV could be assigned to Mo 3d doublets. The fitted Mo 3d peaks positioned at 229.2 and 232.5 eV were corresponding to Mo (IV). While, the doublet peaks at 232.8 and 235.9 eV were indexed to Mo (VI). (Zheng et al., 2017; Lian et al., 2022). As displayed in Figure 2D, the binding energy for Zn 2p3/2 and 2p1/2 were 1,021.8 and 1,044.8 eV, respectively. (Hua et al., 2019).

### 3.2 The peroxidase mimetic activity of Mo@ZIF-8

**FIGURE 3:**
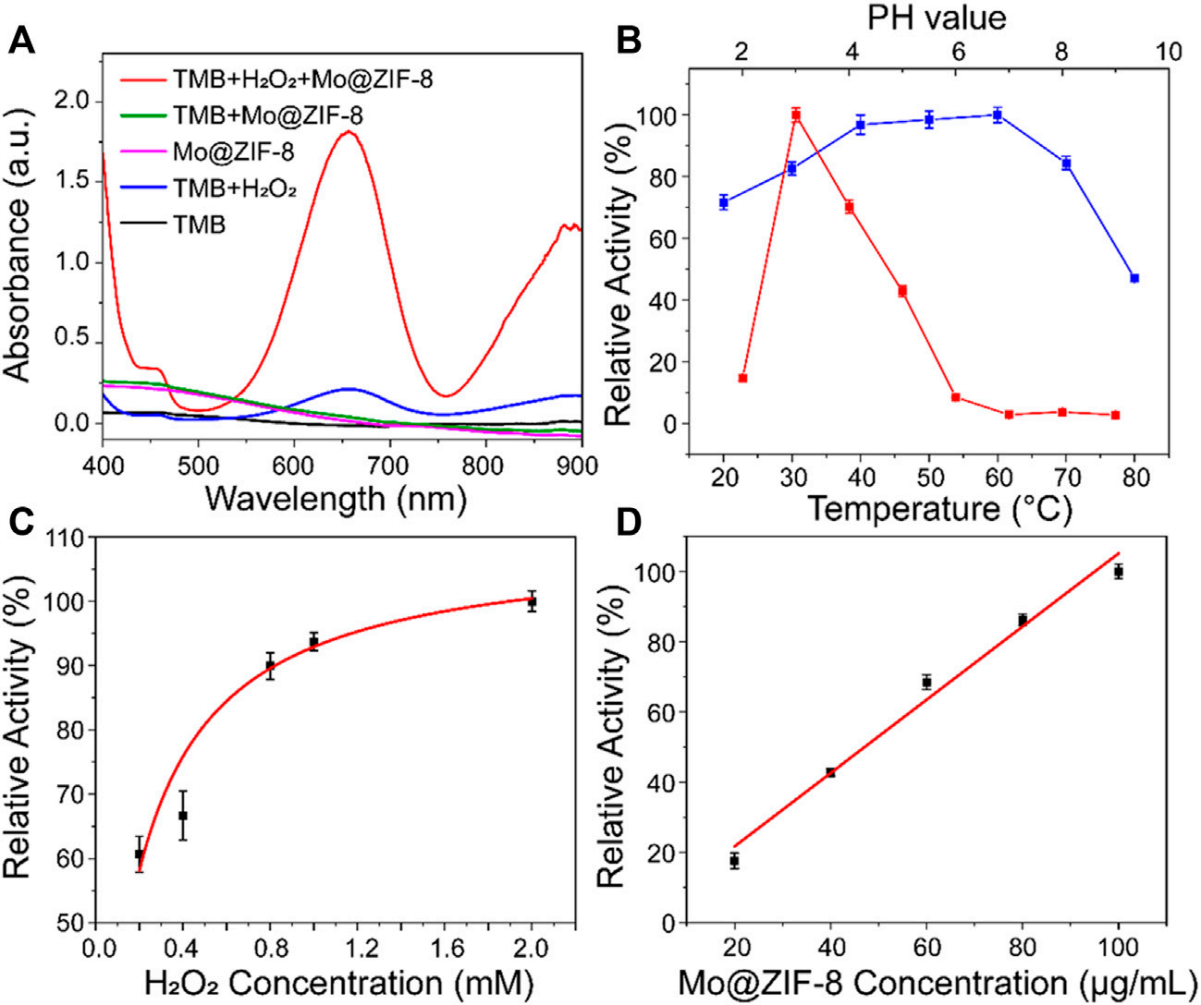
The peroxidase-like activity of the Mo@ZIF-8. **(A)** Absorbance spectra of TMB in diﬀerent systems. **(B)** pH (red) and temperature (blue) dependence of Mo@ZIF-8 peroxidase activity. **(C)** H_2_O_2_ concentration dependence of the peroxidase-like activity of Mo@ZIF-8. **(D)** Concentration dependence of peroxidase activity of Mo@ZIF-8. In **(B–D)**, data are presented as mean ± SD (*n* = 3).

TMB, a classical chromogenic substrate, was used to test the peroxide-like activity of Mo@ZIF-8 nanocomposites in the presence of H_2_O_2_. (Wang et al., 2019). As shown in Figure 3A, Mo@ZIF-8 exhibited highest absorbance at 652 nm, illustrating the high peroxidase-like activity. However, ZIF-8 showed negligible enzymatic activity under the same condition, (Supplementary Figure S2), which indicated that the introduction of Mo element played a key role during the catalytic process. In contrast, H_2_O_2_ alone did not show significant absorption at 652 nm. The catalytic performance of enzyme was highly dependent on pH, temperature, concentration of H_2_O_2_ and nanozyme. Experiments were carried out at diﬀerent pH (2–9) and temperature (20–80°C), and the optimum condition for the peroxide-like activity of Mo@ZIF-8 was found to be pH 3 and 60°C. (Figure 3B). Notably, Mo@ZIF-8 exhibited high catalytic activity in a broad temperature range, illustrating its low sensitivity towards temperature. Furthermore, the catalytic activity of Mo@ZIF-8 was directly enhanced by the increasing concentration of H_2_O_2_ (Figure 3C). And there was almost a linear relationship between the concentration of Mo@ZIF-8 and its relative catalytic activity (Figure 3D).

**FIGURE 4:**
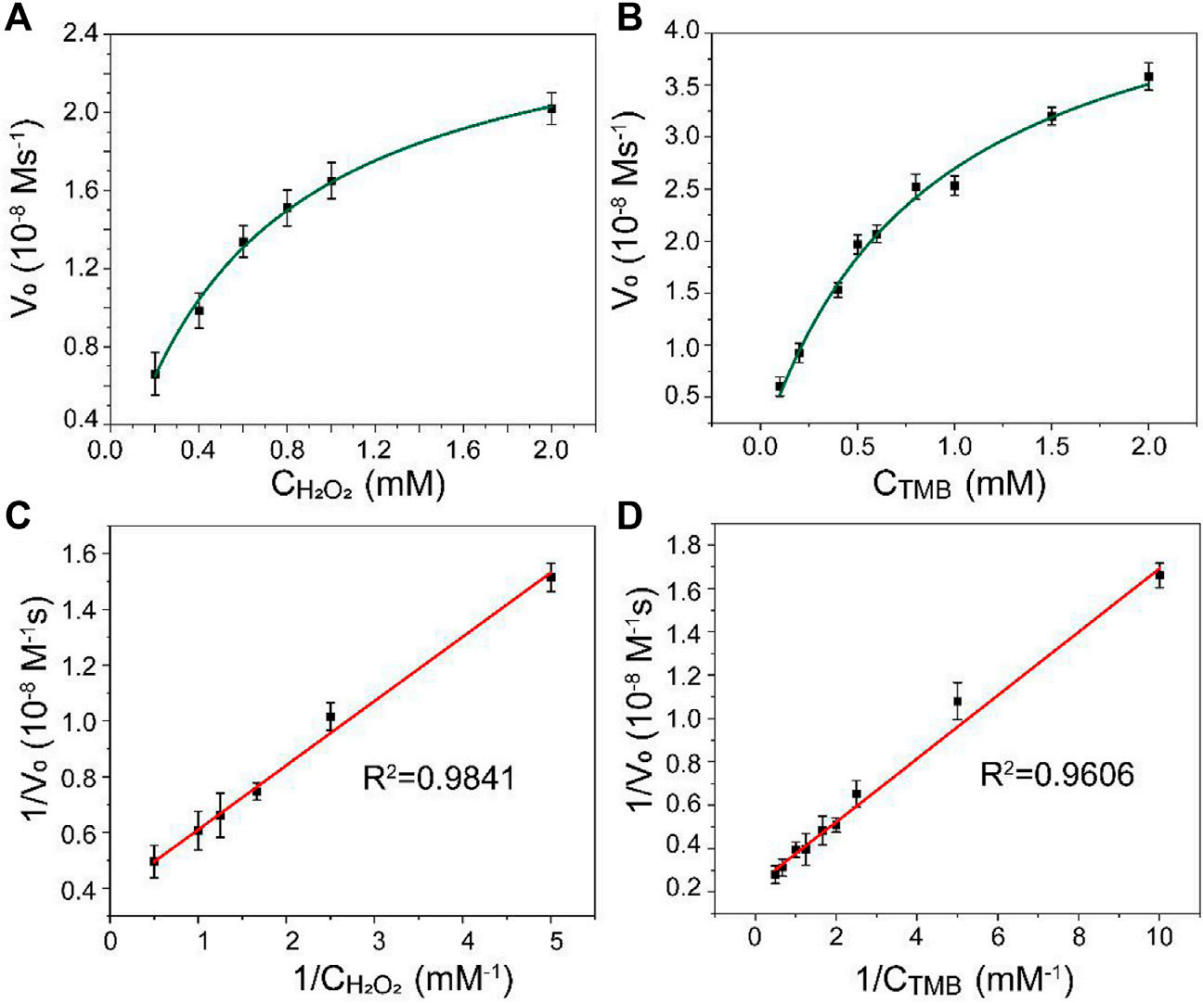
Steady-state kinetic analysis for Mo@ZIF-8-catalyzed TMB oxidation. **(A,B)** Michaelis−Menten model analysis. **(C,D)** Lineweaver−Burk models analysis. Data are presented as mean ± SD (*n* = 3).

The kinetic analysis of Mo@ZIF-8 was further investigated using steady-state kinetic experiments. The data were collected by using a series of TMB concentrations with constant H_2_O_2_ concentration and *vice versa*. The Michaelis–Menten constant (K_m_) and the maximum initial velocity (V_max_) could be calculated from Lineweaver–Burk double reciprocal plots, which showed a good linear-ship between v^−1^ and [S]^−1^. (Figure 4). The K_m_ values of Mo@ZIF-8 were 0.62 and 0.86 mM with H_2_O_2_ and TMB as the substrates, respectively, and the corresponding V_max_ values were 26.64 nM s^−1^ and 50.15 nM s^−1^.

The mechanism of Mo@ZIF-8 peroxidase-like activity was investigated. Assuming that Mo@ZIF-8 could convert H_2_O_2_ into hydroxyl radicals (•OH) through the POD-like activity, terephthalic acid (TA) was used to confirm the production of •OH during the catalytic progress. TA was a nonfluorescent molecule, but it could easily react with •OH to generate highly fluorescent 2-hydroxy terephthalic acid. (Wang et al., 2018). As shown in Supplementary Figure S3, the enhancement in fluorescence intensity was significant when compared with the control group, confirming the production of hydroxyl radicals by Mo@ZIF-8 in the presence of H_2_O_2_. All the results indicated that Mo@ZIF-8 showed POD enzyme mimicking activity and was suitable and efficient for killing bacteria.

### 3.3 Antibacterial experiments

**FIGURE 5:**
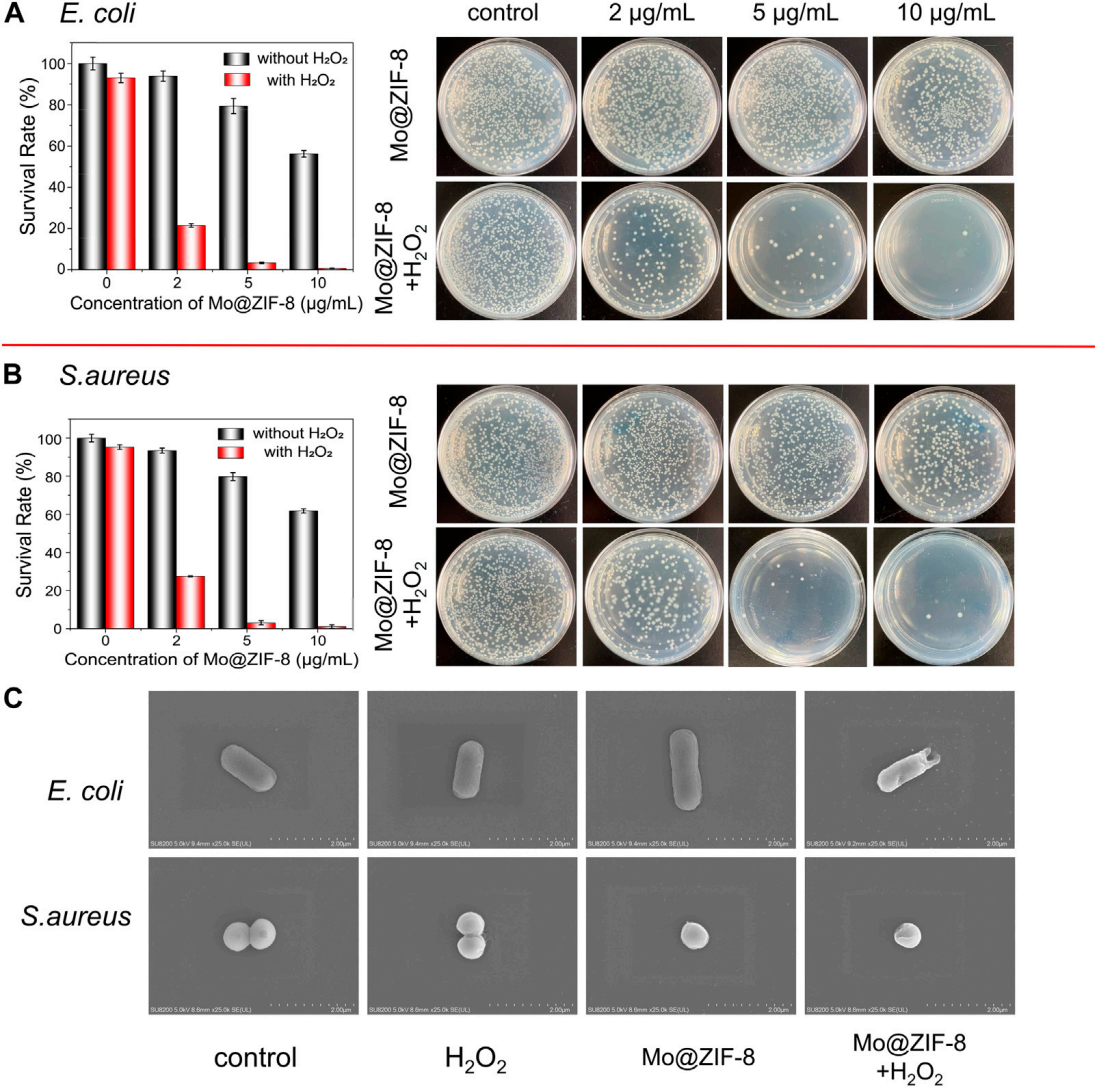
Antibacterial capability of Mo@ZIF-8. Bacterial proliferation and the corresponding images of bacterial colonies on LB agar at different concentration with and without H_2_O_2_. **(A)** Antibacterial capability of Mo@ZIF-8 to *E. coli* (Gram-negative). **(B)** Antibacterial capability of Mo@ZIF-8 to *S. aureus* (Gram-positive). In **(A)** and **(B)**, data are presented as mean ± SD (*n* = 3). **(C)** Representative morphologies of bacteria in different groups imaged by SEM.

The antibacterial effect of the Mo@ZIF-8 nanocomposites was evaluated with the assistance of H_2_O_2_. The bacterial viability was measured by counting the colonies forming units. During the antibacterial experiments, the concentration of H_2_O_2_ was 10^−5^ M, which hardly affect the survival of both *E. coli* and *S. aureus*. Figure 5 showed the images of bacteria colonies on LB agar with various treatments. As shown, Mo@ZIF-8 alone showed a dose-dependent inhibition effect. However, Mo@ZIF-8 combined with low concentration H_2_O_2_ (10^−5^ M) exhibited excellent antibacterial effect, and the inhibition efficiency towards *E. coli* and *S. aureus* reached to 99.2% and 99.4%, respectively, when the concentration of Mo@ZIF-8 was 10 μg/mL. These results illustrated that Mo@ZIF-8 could be used as an efficient antibacterial agent.

The morphological changes of *E. coli* and *S. aureus* were investigated after various treatment by SEM. In Figure 5C, as for *E. coli*, no obvious morphological change was observed in the control, H_2_O_2_ and Mo@ZIF-8 groups, and *E. coli* cells showed rod-shape, with intact and smooth cell walls. As for the groups with treatment of both Mo@ZIF-8 and H_2_O_2_, *E. coli* cells lost their cellular integrity, with noticeable holes on its cell wall. For *S. aureus*, the results were similar. *S. aureus* in the control, H_2_O_2_ and Mo@ZIF-8 groups exhibited a sphere shape, a well-defined and even cell wall. Nevertheless, the morphology of *S. aureus* cells incubated with Mo@ZIF-8 and H_2_O_2_ were changed. In this group, *S. aureus* cells were damaged, even with intracellular components leaked. These results demonstrated excellent antibacterial capability of Mo@ZIF-8 with the assistant of H_2_O_2_.

## 4 Conclusion

In summary, we fabricated a new nanozyme, Mo@ZIF-8 nanocomposites through refluxing Na_2_MoO_4_ solution with ZIF-8 and pyrolyzing at 600°C. This nanocomposite exhibited peroxide-like activity and achieved a wide range of antibacterial capability against both Gram-negative (*E. coli*) and Gram-positive (*S. aureus*) bacteria through producing •OH. Due to the high biocompatibility of the selected components, Mo and ZIF-8, the Mo@ZIF-8 nanozyme has the potential to be an effective antibacterial agent applied in biomedical field.

## Data Availability

The original contributions presented in the study are included in the article/Supplementary Material, further inquiries can be directed to the corresponding author.
