# Supplementary material for: Mo@ZIF-8 nanozyme preparation and its antibacterial property evaluation
Source: Front Chem. 2022 Nov 24;10:1093073. doi: 10.3389/fchem.2022.1093073 (PMC9730516; doi:10.3389/fchem.2022.1093073)
Supplement: Supplementary file 1 [file DataSheet1.docx]

Supplementary Material

## Supplementary Figures


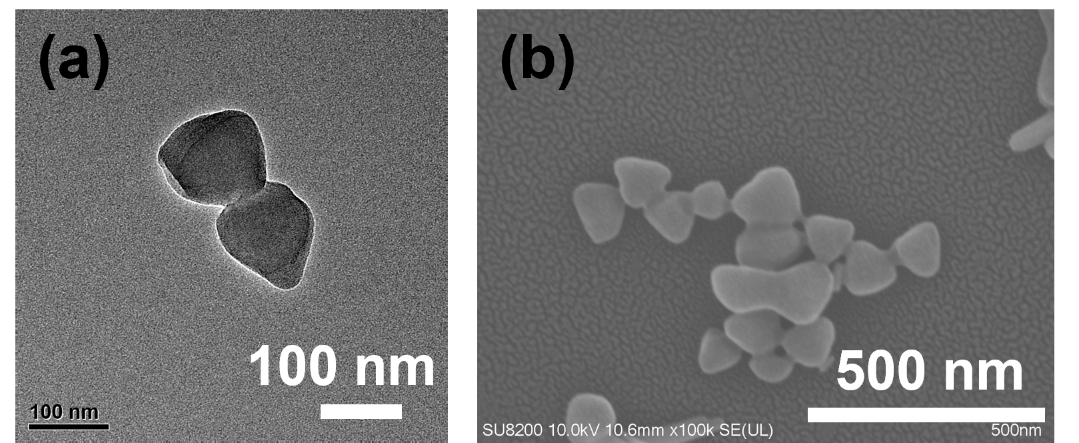


**Supplementary Figure 1.** Morphologies of ZIF-8 after refluxing with Na_2_MoO_4_. (a) TEM image and (b) SEM image.


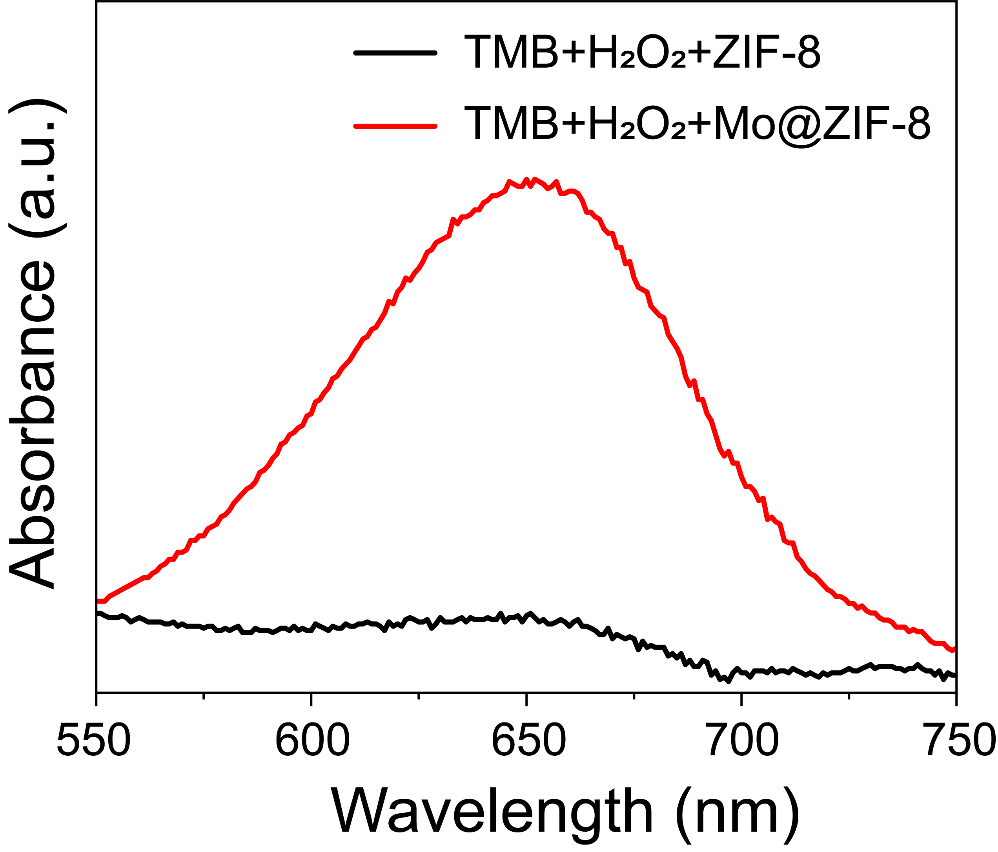


**Supplementary Figure 2.** The peroxidase-like activity of the ZIF-8 and Mo@ZIF-8.


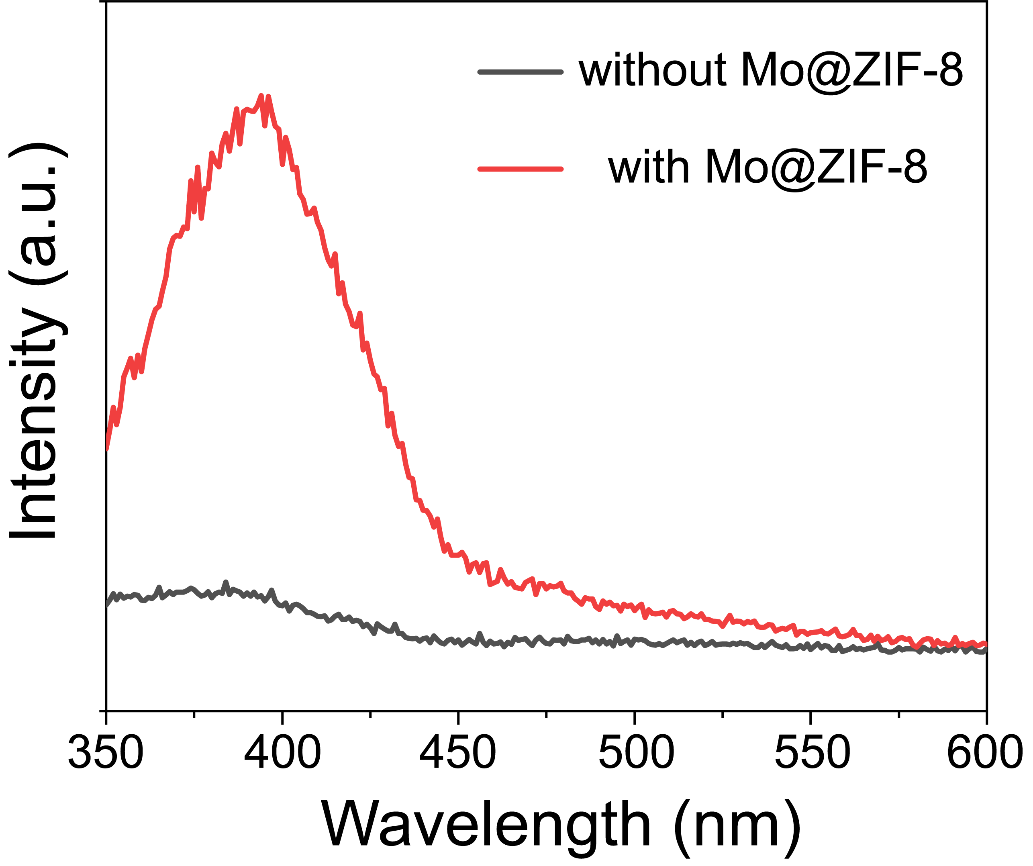


**Supplementary Figure 3.** Fluorescence spectra of the mixture containing TA with or without Mo@ZIF-8 after a reaction time of 30 min.
